# Comparison of the SF6D, the EQ5D, and the oswestry disability index in patients with chronic low back pain and degenerative disc disease

**DOI:** 10.1186/1471-2474-14-148

**Published:** 2013-04-26

**Authors:** Lars G Johnsen, Christian Hellum, Øystein P Nygaard, Kjersti Storheim, Jens I Brox, Ivar Rossvoll, Gunnar Leivseth, Margreth Grotle

**Affiliations:** 1 Neuroclinic; National Center of spinal disorder, St. Olavs Hospital, Trondheim University Hospital, Trondheim, Norway; 2 Clinic of Orthopedics and Rheumatology, Department of Orthopaedic Surgery, St. Olavs Hospital, Trondheim University Hospital, Trondheim, Norway; 3 Department Of Neuromedicine, Faculty of Medicine, Norwegian University of Science and Technology, Trondheim, Norway; 4 Clinic for Surgery and Neurology, Department of Orthopedics, Oslo University Hospital and University of Oslo, Oslo, Norway; 5 Department of Clinical Medicine, Neuromuscular Diseases and Research Group, University of Tromsø, Tromsø, Norway; 6 Clinic for Surgery and Neurology, Oslo University, Oslo, Norway; 7 Department of neurosurgery, St. Olavs Hospital, Trondheim University Hospital, Trondheim, Norway; 8 FORMI, Clinic for surgery and neurology, Ullevaal, Oslo, N-0407, Norway; 9 Faculty of health Sciences, Department of Physiotherapy, Oslo and Akershus University College of Applied Sciences, Oslo, Norway

**Keywords:** Utility measures, Outcome assessment, Measurement properties, Health economics, Low back pain, Lumbar disc prosthesis, EQ5D, SF6D

## Abstract

**Background:**

The need for cost effectiveness analyses in randomized controlled trials that compare treatment options is increasing. The selection of the optimal utility measure is important, and a central question is whether the two most commonly used indexes - the EuroQuol 5D (EQ5D) and the Short Form 6D (SF6D) – can be used interchangeably. The aim of the present study was to compare change scores of the EQ5D and SF6D utility indexes in terms of some important measurement properties. The psychometric properties of the two utility indexes were compared to a disease-specific instrument, the Oswestry Disability Index (ODI), in the setting of a randomized controlled trial for degenerative disc disease.

**Methods:**

In a randomized controlled multicentre trial, 172 patients who had experienced low back pain for an average of 6 years were randomized to either treatment with an intensive back rehabilitation program or surgery to insert disc prostheses. Patients filled out the ODI, EQ5D, and SF-36 at baseline and two-year follow up. The utility indexes was compared with respect to measurement error, structural validity, criterion validity, responsiveness, and interpretability according to the COSMIN taxonomy.

**Results:**

At follow up, 113 patients had change score values for all three instruments. The SF6D had better similarity with the disease-specific instrument (ODI) regarding sensitivity, specificity, and responsiveness. Measurement error was lower for the SF6D (0.056) compared to the EQ5D (0.155). The minimal important change score value was 0.031 for SF6D and 0.173 for EQ5D. The minimal detectable change score value at a 95% confidence level were 0.157 for SF6D and 0.429 for EQ5D, and the difference in mean change score values (SD) between them was 0.23 (0.29) and so exceeded the clinical significant change score value for both instruments. Analysis of psychometric properties indicated that the indexes are unidimensional when considered separately, but that they do not exactly measure the same underlying construct.

**Conclusions:**

This study indicates that the difference in important measurement properties between EQ5D and SF6D is too large to consider them interchangeable. Since the similarity with the “gold standard” (the disease-specific instrument) was quite different, this could indicate that the choice of index should be determined by the diagnosis.

## Background

An important way of assessing the effects of treatment in health economic evaluations is the use of utility indexes. The outcome scores of general health-related quality of life (HRQoL) questionnaires are stratified into different health states [1,2] that can then be validated in a community population [3,4]. Treatment benefit is thus expressed in a way that allows health states that are considered less preferable (0) to full health (1) to be given quantitative values. Because these quantitative values represent a valuation or preference of health states for the patients, they are called utility indexes (more utility for the patient with increasing value). When combined with a follow up period, health utility indexes are used to calculate quality-adjusted life years (QALYs). There are several utility indexes that could be used, and discrepancies exist regarding which index is most suitable [1,5]. These discrepancies could have implications for calculating cost-effectiveness when comparing alternative treatment options for the same disease [6-10]. Two of the most widely used indexes are the EuroQuol 5D (EQ5D) and the Short Form 6D (SF6D) [4,7].

Two papers assessed the impact that the measure has on cost-utility estimates [8,9]. Sach et al. found that the SF6D and EQ5D favored different treatment options for alleviating knee pain when applying the same cost per QALY threshold. Søgaard et al. [11] reported on the interchangeability of the two indexes. When plotting difference between change scores of SF6D and EQ5D against their average in a Bland-Altman plot , they found that the expected between-measure variation was 0.546 [12]. They conclude that although both indexes appear to be psychometrically valid for generic assessment of long-lasting back pain, the variation between them was too great to be considered interchangeable.

From other studies, we could hypothesize that there would be a discrepancy between the EQ5D and SF6D because of differences in valuing similar health states, evidence of a floor effect in the SF6D and a ceiling effect in the EQ5D, and because the SF6D can describe severe health states better than EQ5D [7,13,14].

Further work is required in this field to understand these discrepancies. Therefore, the aim of this study was to evaluate change scores values of the EQ5D and SF6D utility indexes in terms measurement error, structural validity, criterion validity, responsiveness, and interpretability according to the COSMIN taxonomy. The psychometric properties of the two utility indexes were compared to a disease-specific instrument, the Oswestry Disability Index (ODI), in the setting of a randomized controlled trial for degenerative disc disease.

## Methods

Details about the RCT on which this work is based is reported in detail in Hellum et al. [15]. Between April 2004 and September 2007, 172 patients with diagnosed chronic low back pain and degenerative disc disease were randomized to either surgery with total disc replacement or multidisciplinary rehabilitation. The results from this study have been published previously [15].

Briefly, data were collected in a multicentre randomized controlled trial involving the five university hospitals in Norway. Inclusion criteria included age between 25 and 55 years, LBP for more than a year, degenerative changes in the intervertebral disc in one of the two lowest levels of the lumbar spine and an Oswestry Disability Index score of 30% points or more. Exclusion criteria included generalized chronic pain syndrome and degeneration established in more than two levels. Part of this study was an economic evaluation of chronic low back pain treatment. Patients were randomized to either surgery with insertion of an artificial disc or to non-surgical treatment (a multidisciplinary back rehabilitation program).

The outcomes of patients who completed the SF6D, EQ5D, and ODI at baseline and at 2-year follow up were included in this study.

### Instruments

#### ODI

The ODI is a back-specific questionnaire [16,17]. Patients rate physical disability in activities of daily living due to low back pain in 10 questions, each of which has verbal response alternatives. Ratings are summed to yield a score ranging from 0 (not disabled at all) to 100 (completely disabled). We used the Norwegian translation of the validated questionnaire (version 2.0) [18].

#### SF6D

The SF6D utility index is comprised of 11 items from the SF-36 [19] that were revised into a six-dimensional health state classification system. The six dimensions are physical functioning, role limitations, social functioning, pain, mental health, and vitality. It reflects a continuous outcome scored on a 0.29–1.00 scale, with 1.00 indicating full health [3]. SF6D health states were evaluated against a normal population using the Standard Gamble (SG) method. We used the United Kingdom (UK) tariff [3]. The SF6D was calculated based on the Norwegian SF-36 (version 2) with the use of syntax files in SPSS 17(SPSS, New York, US). The syntax files were kindly provided by Dr J. Brazier, University of Sheffield, UK.

#### EQ5D

For the EQ5D utility index, responses on a questionnaire with five dimensions, each comprised of three levels, are revised into an index with a range from −0.59–1, with 1.00 indicating full health. The 243 possible health states on the EQ5D are evaluated against a normal population using the time trade off method (TTO) [20,21]. We used the Norwegian version of the EQ5D and syntax files obtained from the EQ5D society using the UK tariff to calculate the index.

#### Seven-point scale for patient assessment

Many authors suggest a seven-point scale to assess patient outcome in terms of a global score [22]. On the question: “How much benefit do you think you have had from the treatment you have received?” patients answered on a 7-category response scale that ranged from “I am completely disabled” to “I am completely recovered”.

### Data analysis

We followed the definitions and recommendations from The COSMIN (COnsensus-based Standards for the selection of health Measurement INstruments) checklist when analyzing the psychometric properties of the two utility indexes and ODI in this study [23].

If not otherwise mentioned, SPSS version 17 was used in the statistical analysis.

### Measurement error

Measurement error concerns the systematic and random error of a patient`s score that is not attributed to true changes in the construct to be measured [24]. We used the standard error of measurement (SEM) to express instrument imprecision [22,25-27]. The advantage of using SEM is that it is considered to be an attribute of the measure and not a characteristic of the sample itself [28]. The SEM value could be calculated from a test-retest study or in a group of stable patients. The SEM in this study was calculated as:

$$S_{w}=SEM=\sqrt{\frac{1}{2n}\sum d_{t}^{2}}$$

where s_w_ is the within-subject standard deviation, d is the difference between two observations in patients *i* who reported “unchanged” on a four-point scale between 3 and 6 months follow up and n is the number of subjects [29]. The s_w_ statistics is also called the SEM_consistency_[30].

The lowest change that exceeds measurement error and noise at a 95% confidence level is defined as:

$$MDC_{95}=1.96*\sqrt{2}*SEM=2.77*SEM$$

Here, the * $\sqrt{2}$ is introduced because there are two measurements for each patient. The minimum detectable change (MDC) at a 95% confidence level, is denoted MDC_95_[31]. With a scale value ≥MDC_95_, we can be 95% certain that a change in the measured underlying construct has really occurred [32].

To assess the agreement between EQ5D and SF6D, a Bland Altman plot was constructed. [12]. The average EQ5D and SF6D change score values were plotted against the mean difference in change score values of both instruments. Limits of Agreement (LoA) based on a +/− 1.96*SD_difference_ interval for the differences were also constructed.

### Structural validity

Structural validity concerns the degree to which the scores of an instrument are an adequate reflection of the dimensionality of the construct to be measured [33]. Both EQ5D and SF6D are constructed to measure the dimension of general health related quality of life (HRQoL) alongside a continuous scale (from low to high). Using Item Response Theory (IRT), the unidimensionality of the two utility indexes was tested. The category ordering of the questionnaire items (the probability of moving from an easier to a harder accomplished category of item answers in parallel with being increasingly disabled) was also tested.

We employed the unrestricted (Partial-Credit) polytomous model of the Rasch model (for general information about fit to the Rasch model, see Additional file 1) and the test proposed by Smith to reveal unidimensionality [34]. The SF6D and EQ5D were tested for unidimensionality in a principal component analysis (PCA) [35]. We performed a test equating procedure with baseline values from the SF6D and the EQ5D. The response of each patient to a question was tested against what was predicted by the Rasch model. Deviation from the model is expressed in residuals. Independent t-tests were used to test if the magnitude of the residuals represents a significant deviation. The CI calculated for this was 95%. We carried out a binominal test for the proportion of t-tests outside the range of −1.96–1.96. The software used in the Rasch analysis was RUMM 2020 (RUMM Laboratory Pty Ltd.).

### Criterion validity

Criterion validity concerns the degree to which the scores of an instrument are an adequate reflection of a “gold standard” when this is present [33]. In this analysis we compared the scores of the EQ5D and SF6D to the disease specific instrument ODI. The rationale was that the ODI has been found to be a responsive and valid measure for patients with LBP [16,18,36] and that an improvement assessed by the ODI should be correlated with an improvement assessed by the two utility indexes.

Spearman rank correlation coefficient (r) with 1000 bootstrap replications of the *baseline* scores was calculated to assess the correlation between the scores of the EQ5D and ODI and SF6D and ODI.

### Responsiveness

Responsiveness is defined as the ability of an instrument to detect change over time in the construct to be measured [33]. Responsiveness was assessed by using the ODI and the seven-point global scores at 2-year follow-up as “gold standard”. First, we calculated the Spearman rank correlation coefficient (r) with 1000 bootstrap replications for the correlation between *change* scores from baseline to 2 year FU for the EQ5D, SF6D and ODI. Second, we analyzed the area under the Receiver Operator Curve (ROC) for the change scores of the EQ5D, SF6D and ODI by using a dichotomization of the patient global scores as follows: Categories 1 to 3 was considered “improved” and categories 4 to 7 were “non-improved”. Sensitivity was defined as the proportion of patients who were correctly classified as “improved” and specificity was defined as the proportion of patients who were correctly classified as “non-improved”. A receiver operating characteristic (ROC) curve was then calculated by plotting every possible change score from baseline to 2 year FU for EQ5D, SF6D and ODI using the global score as an anchor [37,38]. The area under the ROC curve (AUC) was then calculated. This value corresponds to the possibility of correctly diagnosing a patient as having improved when this is really the case [38] and reflects how responsive the instruments are to detect a change in the underlying construct.

The calculation of ROC curves was performed with MedCalc Statistica software (version 11.1.1. for Windows, Brussels, Belgia).

### Interpretability

Interpretability concerns the qualitative meaning of quantitative scores or change in scores. A core question is: “What is the smallest change in score in the construct to be measured which patients consider important? This is expressed as the Minimal Important Change (MIC) value [33], and is calculated based on the sensitivity and specificity results from the ROC analysis described above. The cut-off value for differentiating between patients with or without improvement at optimum sensitivity and specificity was determined using ROC analysis [38]. This corresponds to the upper left point on the ROC curve and it can be interpreted as the point or value that yields the lowest overall misclassification [25,39].

### Study approval

The study was evaluated and approved by the regional Committee for Medical Research Ethics in east Norway. Storage of data was allowed by the Norwegian Data Inspectorate. The study was conducted in accordance with the Helsinki Declaration and the ICH-GCP guidelines and registered at clinicaltrial.gov under the identifier NCT00394732.

## Results

At inclusion, there were 52,6% females. Mean age was 41 years and mean (SD) duration of low back pain was 6 years (5,74). Response rates at baseline and 2-year follow up and pre- and post-treatment scores are presented in Table 1. At baseline, 133 out of 173 patients had completely filled out the ODI, the EQ5D, and the SF-36, so values for each of the instruments could be calculated. At 2-year follow up, 113 patients had values for all three instruments, so change scores could be calculated.

**Table 1:** Response rate at baseline and two year follow up together with pre- and post-treatment scale scores

	**Response rate**		**Mean scale score (SD)**	
	**Baseline**	**2 years**	**Baseline**	**2 year**
ODI	99%	100%	42,29 (0,81)	23 (16)
SF6D	82%	90%	0.555 (0,007)	0.692 (0.143)
EQ5D	93%	99%	0.292 (0.026)	0.642 (0.318)

N = 133.

### Measurement error

The SEM values calculated for patients who were stable for a period of 3 months are presented in Table 2.

**Table 2:** **SEM and MDC**_**95 **_**values**

	**SEM**	**MDC**_**95**_
ODI	4.24	11.75
SF6D	0.056	0.157
EQ5D	0.155	0.429

The SEM represents the standard error of measurement. The MDC_95_ is the minimal detectable change value that falls outside the measurement error of the instrument with 95% probability.

The smallest change score that could be said to represent a real change beyond measurement error with 95% probability in one individual (MDC_95_) are presented in Table 2.

The proportion of patients with a change score value ≥MCD_95_ was 69% for ODI, 57% for SF6D, and 45% for EQ5D.

**Figure 1:**
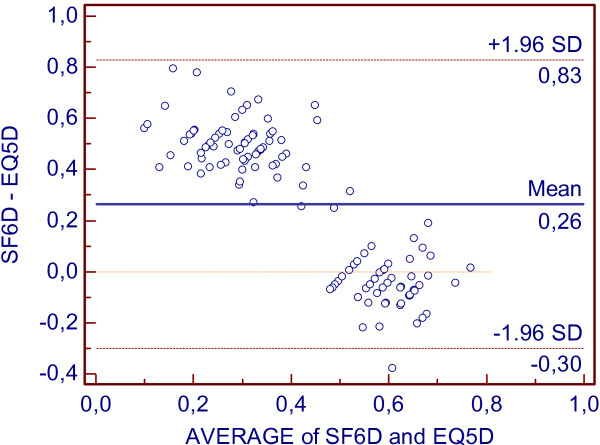
Bland-Altman plot.

Figure 1 shows a Bland-Altman plot of the SF6D and EQ5D baseline values. It illustrates a systematic variation (proportional error) in the EQ5D and SF6D scores, with less healthy individuals tending to have a higher score on the SF6D and healthier individuals tending to have a higher score on the EQ5D. The 95% Limits of Agreement (LOA) varied from −0.3 to 0.83 with a mean difference in scale scores (SD) of 0.23 (0.29).

### Structural validity

When the SF6D items were used as one subset and the EQ5D items as another, the binominal test showed overlap of the 5% expected value with the 95% CI for each of the indexes. When the EQ5D and SF6D items were combined on a common scale, no overlap was identified. This finding could indicate that the indexes are unidimensional when considered separately, but that they do not exactly measure the same underlying construct [34,40].

**Figure 2:**
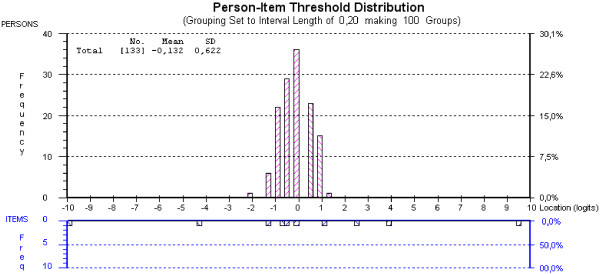
Person-item threshold distribution for EQ5D.

**Figure 3:**
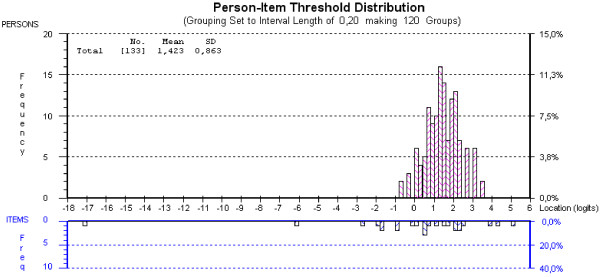
Person-item threshold distribution for SF6D.

Figures 2 and 3 are graphic representations of the targeting of the SF6D and EQ5D items. Patients “ability” (level of health-related quality) and the item location (moving from an easy to a more difficult category of item answers in parallel with being increasingly disabled) are plotted on the same logarithmic scale. The bars in the top panels represent patient responses, and the bars in the bottom panels represent item thresholds on the scales. A threshold is the 0.5 probability point between adjacent item categories [41]. HRQoL levels (i.e., scoring values) decrease from left to right. Scoring responses outside the range of items represent a floor effect (to the right) or a ceiling effect (to the left). Responses outside the range of the scale give no additional information, and the test cannot discriminate between patients who fall in this area.

From Figures 2 and 3 it can be seen that the EQ5D was relatively well targeted for this group, with no sign of floor or ceiling effects, i.e., all responders were captured within the scale. With a m**e**an person-location value of −0.132, the patients were at a slightly higher level of HRQoL than the scale could express. No floor or ceiling effect could be seen in the SF6D, but here the mean person-location was 1.423. This indicates that there is a tendency for patients to score at the lower end of the scale of this index.

Three of the items in the SF6D showed disordered threshold: question 1: Physical functioning, question 2: Role limitation and question 4: Pain. A better fit to the model was achieved if some of the response categories of these items were omitted. None of the questions in the EQ5D showed disordered thresholds.

### Criterion validity

The correlation between *baseline* scores of ODI and EQ5D was r = 0,58 (n = 114, p=0.000) and for ODI and SF6D: r = 0.38 (n = 114, p = 0.000).

### Responsiveness

**Figure 4:**
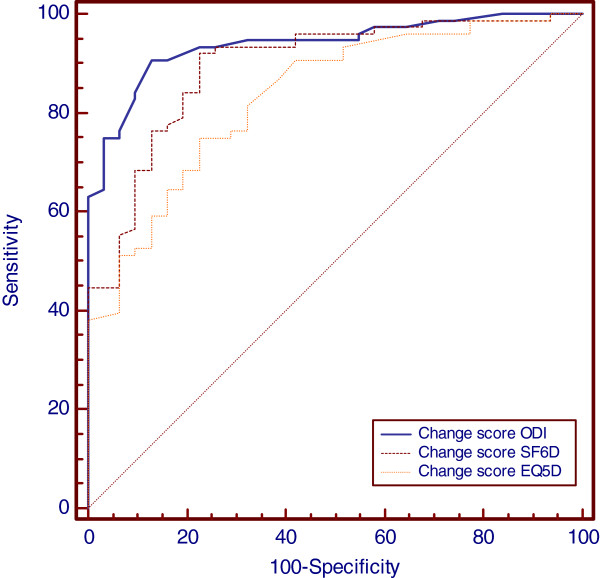
ROC curve.

a) The correlation between *change* scores of ODI and EQ5D was r = 0,64 (n = 108, p=0.000) and between ODI and SF6D change scores: r = 0.77 (n = 108, p = 0.000).

b) Spearman’s rho for the correlation between change scores of the instruments and global score categories was 0.84, 0.55 and 0.76 for ODI, EQ5D and SF6D respectively. The area under the ROC curve, the possibility of correctly discriminating between “improved” or “non-improved” patients with a 95% CI was: 94% (87.5–97.6) for ODI, 90% (82.1–94.6) for SF6D, and 83% (75–90) for EQ5D. The ROC curves are presented in Figure 4.

### Interpretability

The MIC values defined as the most optimal cut-off point of change scores plotted on the ROC curve was for ODI: 12.88,(sensitivity 88%, specificity 85%), EQ5D: 0.173 (sensitivity: 73%, specificity 79%) and SF6D: 0,031 (sensitivity 93%, specificity 78%) (Figure 4).

## Discussion

The present study failed to show similarity between EQ5D and SF6D in several important measurement properties. EQ5D had a higher value of inherent measurement error than SF6D. The mean difference between baseline score values had a wide 95% Limits of Agreement in the Bland-Altman plot signifying a low degree of agreement between the instruments [12,42]. Rasch analysis showed that although EQ5D and SF6D separately seem to have unidimensional scale properties they probably do not measure the same underlying construct. SF6D show less similarity with the baseline scores of the disease specific instrument but were more responsive to detect a change in the underlying construct in addition to better ability to correctly diagnosing a patient as having improved when this was really the case even though it did not reach the level of the ODI. The MIC values were quite different and SF6D had a better ability to identify truly change in scale score beyond measurement error.

Van Stel et al. showed that the EQ5D and the SF6D yield dissimilar scores in patients with coronary heart disease, and consequently, they cannot be used interchangeably [43]. This is in line with the Bland-Altman plot pattern we found in our study and in agreement with other previously published reports [6,13,43]. Furthermore, we observed that the magnitude of difference between the two instruments in the Bland-Altman plot was beyond the MIC for both instruments and therefore interpreted as clinically significant.

In this study, sensitivity was defined as the proportion of patients that truly improved (true-positive rate), and the sensitivity was the proportion of patients that did not actually improve (true-negative rate). The EQ5D diagnosed fewer patients as clinically improved (change score values beyond MIC). This was also reflected in the MIC/MDC_95_ ratio (the proportion of patients who truly changed with a possibility of 95% predicted by the instruments): For the MIC value to reach the MDC_95_, the specificity for the SF6D would have to increase from 78.1 to 87.5, but the sensitivity would then fall from 92.5 to 73.7. For the EQ5D, this would necessitate an increase in specificity from 78.9 to 86.8 and a decrease in sensitivity from 72.8 to 57.6. In other words, to reach a value beyond the 95% CI for measurement error, the probability of correctly classifying a patient as improved would fall dramatically for the EQ5D, nearly reaching 50% or classifying by chance. The effect was not as dramatic for the SF6D, which would still correctly classify over 70% of patients as “improved”.

We found that the difference in the range of the scales between the SF6D and the EQ5D could be reflected in their targeting properties. Based on the Rasch analysis (Figures 3 and 4), we could hypothesize that patients were at a lower level of HRQoL than the SF6D could express (floor effect). The range of patient abilities was better captured within the EQ5D scale. Barton et al. [6] compared the performance of the EQ5D and the SF6D in 1865 individuals over ≥45 years old. They found that healthier individuals had higher scores on the EQ5D, and less healthy individuals such as patients with back pain had higher scores on the SF6D. In a study that compared the SF6D and the EQ5D in liver transplant patients, Longworth et al. observed that the SF6D does not describe health states at the lower end of the utility scale but is more sensitive than the EQ5D in detecting small changes at the top of the scale [14]. This result is somewhat confusing because the same group later published a paper in which they conclude that the SF6D can describe some “poor health states including states that (according to the EQ5D scoring algorithm) are viewed as worse than the state of being dead” [13].

The Rasch analysis also revealed that some of the SF6D items did not function as intended. A better fit to the model was achieved if some of the response categories of these items were collapsed (i.e., the category was removed from the item). An interpretation of this is that for these items, patients could not differentiate between two adjacent response categories and the information in the removed categories was therefore redundant. None of the items in the EQ5D showed similar signs of dysfunction. When treated as separate scales, both instruments showed signs of unidimensionality, but significant invariance across items was noted when analyzed as one scale (all items from the SF6D and the EQ5D put together). The interpretation of this was that the two scales seem to measure different aspects of HRQoL. Walters and Brazier mentioned that a fundamental assumption in their comparison of the EQ5D and the SF6D was that the instruments should measure the same underlying HRQOL variable [44].

### Strengths and limitations of the study

Compared to Brazier et al. [7], SF6D in our study had a higher percentage of missing data at both assessment time points (baseline and 2-year follow up). As Brazier mentioned in another paper, this has important consequences for data quality [45].

The use of global assessment score has been questioned in several studies [46,47]. Criticism of the reliability of anchor based methods includes no standardization of anchors, time dependence of patients perception of health, dependence on only one question and failure of the anchor question to differentiate between quantitative and qualitative perception of change [48]. The COSMIN study did not reach any consensus about which method to use to determine the MIC value but conclude that there is an ongoing discussion about this in the literature [23]. Some authors now suggest ROC analysis for determining MIC values mainly because it uses all available data and maximizes the number of individuals correctly classified [49]. The question and answer categories in our 7-point global scale was not a standardized scale but Spearman`s rho for the correlation between change scores of the instruments and global score categories used in the ROC analysis was considered acceptable (0.84, 0.55 and 0.76 for ODI, EQ5D and SF6D respectively) [46,50,51].

## Conclusions

EQ5D and SF6D measure different aspects of HRQoL. The difference in psychometric properties between them and the lack of agreement is probably clinically significant. Because the ability to detect a change in the underlying construct and similarity to a disease-specific instrument is quite different, the choice of instrument should probably be guided by diagnosis and/or treatment choice. In our study of patients with chronic low back pain, the SF6D had the best ability to detect change and correctly identify patients as improved or non-improved beyond a 95% confidence level of measurement error.

Finally, our study supports the findings of Soegaard et al. [11]. They concluded that the SF6D and EQ5D cannot be used interchangeably for measurement of preference value and that sensitivity analysis examining the impact of between-measure discrepancy remains a necessary condition for cost-utility evaluation results.

## Competing interests

The authors declare that they have no competing interests.

## Authors’ contributions

LGJ takes responsibility for the integrity of the data and the accuracy of the data analysis. LGJ performed the statistical analysis. LGJ, MG, IR and CH participated in the design of the study. LGJ and CH: Acquisition of data. LGJ, CH, ØPN, KS, JIB, IR, GL and MG conceived of the study and helped to draft the manuscript. All authors had full access to the data. All authors read and approved the final manuscript.

## Authors’ information

LGJ: M.D. orthopaedic surgeon, CH: M.D. orthopaedic surgeon., KS: Ph.D. physiotherapist, ØPN: M.D, Ph.D. neurosurgeon, professor, JIB: M.D., Ph.D. specialist in physical medicine and rehabilitation, Ivar Rossvoll: M.D., Ph.D. orthopaedic surgeon, GL: M.D., Ph.D. specialist in physical medicine and rehabilitation, professor.

## Pre-publication history

The pre-publication history for this paper can be accessed here:

http://www.biomedcentral.com/1471-2474/14/148/prepub

## Supplementary Material

Additional file 1 Rasch analysis. Click here for file
