# Supplementary material for: Comparison of the SF6D, the EQ5D, and the oswestry disability index in patients with chronic low back pain and degenerative disc disease
Source: BMC Musculoskelet Disord. 2013 Apr 26;14:148. doi: 10.1186/1471-2474-14-148 (PMC3648434; doi:10.1186/1471-2474-14-148)
Supplement: Additional file 1 — Rasch analysis. [file 1471-2474-14-148-S1.docx]

**Rasch analysis**

Rasch analysis is a probabilistic model that tests the extent to which the observed pattern of responses fits the pattern expected by the model [[1](#_ENREF_1)] [[2](#_ENREF_2)] [[3](#_ENREF_3)]. The model shows what should be expected in responses to items if measurement (at the metric level) is to be achieved [[4](#_ENREF_4)]. Two qualities are central: the ability of a person and the difficulty of an item. The ability can be any clinical sign, such as low back pain. The difficulty of an item could be seen as a measure of the extent to which a person has the ability (e.g., more or less low back pain). The model states that the probability that a person will affirm an item is a logistic function of the difference between a person’s level of, for example, reduced physical function due to low back pain (θ) and the level of reduced functional level expressed by the item (b) and only a function of that difference [[4](#_ENREF_4)]

P_nij_ is the probability that a person *n* will answer in the “affirm” category *j* of item *i* (or be able to do the level of a task specified by that category within the item). Rasch analysis also offers the possibility of converting ordinal raw data into a linear scale if the data fit the model. We used a polytomous variant of the Rasch model, which is known as the partial credit model [[5](#_ENREF_5)].

In PCA, we explore the relationship of the items to the components that contribute most to the variation in data after the Rasch component is removed[[6](#_ENREF_6)]. This is done by comparing fit residuals for each person for each item using independent t-tests [[7](#_ENREF_7)]. The first component in the PCA is the component that accounts for the most variance in the data and can be seen as a “second dimension.” To examine this, we used the subsets of items that loaded the most strongly on the first component because these were the most likely to breach the assumption of unidimensionality. In other words, if these two subsets showed a significant difference from the overall scale, then the assumptions of unidimensionality could be broken. If the data fit the model, then analysis of any subsets of items should produce equivalent person measures within measurement error.

*Threshold order*

A threshold is defined as the 0.5 probability point between adjacent categories of an item[[4](#_ENREF_4)]. The probability of affirming one category response is illustrated by probability density curves for each of the categories ([fig 5](#Fig5)). The 0.5 probability is then at the top of the curve. For categories in increasingly or decreasingly order the top of curve 1 should come before curve 2, the top of curve 2 should come before curve 3 etc. When this is not the case, the thresholds are disordered ([fig 6](#Fig6)). For SF6D, disordered thresholds were found between category 3 and 4 in “Physical”, between category 2 and 3 in “Role” and between category 1 and 2 in “Pain”.

**Fig. 5****. Example of item with ordered thresholds**

**
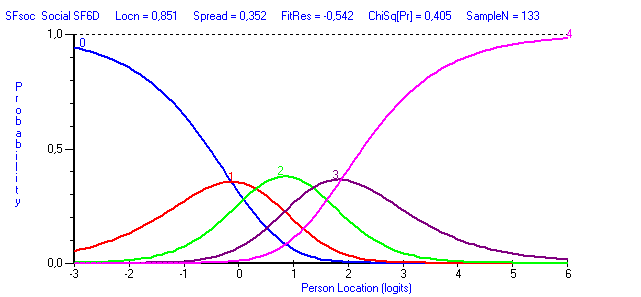
**

**Fig. 6** **Example of item with disordered thresholds**

**
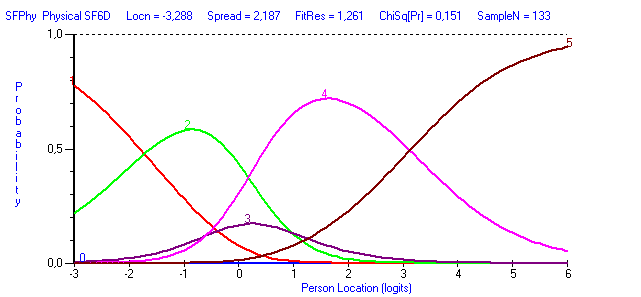
**

1. Rasch G: **Probabilistic models for some intelligence and attainment tests**. Chicago: University of Chicago; 1960.

2. Davidson M: **Rasch analysis of three versions of the Oswestry Disability Questionnaire**. *Man Ther* 2007.

3. Rasch G: **An item analysis which takes individual differences into account**. *Br J Math Stat Psychol* 1966, **19**(1):49-57.

4. Pallant JF, Tennant A: **An introduction to the Rasch measurement model: an example using the Hospital Anxiety and Depression Scale (HADS)**. *Br J Clin Psychol* 2007, **46**(Pt 1):1-18.

5. **A rasch model for partial credit scoring**. *Psychometrika* 1982, **47**(2):149-174.

6. Chou Y-T, Wang W-C: **Checking Dimensionality in Item Response Models With Principal Component Analysis on Standardized Residuals**. *Educational and Psychological Measurement* 2010, **70**(5):717-731.

7. Smith EV, Jr.: **Detecting and evaluating the impact of multidimensionality using item fit statistics and principal component analysis of residuals**. *J Appl Meas* 2002, **3**(2):205-231.
